# The default state of the cell: Quiescence or proliferation?

**DOI:** 10.1002/bies.201100138

**Published:** 2012-01

**Authors:** Edward Parr

**Affiliations:** UBC Envision GroupSouthport, CT, USA

There exists abundant evidence that mutations that change the function or expression of growth-regulatory genes can lead to progression to a cancerous phenotype, and the progressive accumulation of such changes as the underpinning of tumorigenesis forms the basis for the somatic mutation theory (SMT) of cancer [1]. At a molecular level, that model of tumorigenesis suggests that progression to a cancerous phenotype can be driven by gain-of-function in growth-promoting oncogenes and/or loss-of-function in growth inhibitory tumor suppressor genes within somatic cells (a third class of “stability” genes are also a contributor to tumorigenesis [2] but are not considered in this paper). A frequently used analogy for that model compares these classes of genes to the gas pedal and brakes of an automobile [2]. However, there is evidence that this model may not be sufficient in itself to explain the development of cancer [3]. For example, the influence of the milieu of tumor development may strongly influence its development, and so an alternative model in which the tissue microenvironment is a critical factor in tumorigenesis has provided the basis for the tissue organization field theory (TOFT) [4]. Although not all differences between these theories are irreconcilable, a key difference between these models is that quiescence is postulated to be the default state of “normal cells” in SMT, whereas proliferation is assumed as the default state of cells in TOFT [4].

It follows from the requirement for growth promotion in the SMT that the default state of the cell is static. Indeed, it is well established that primary cultured cells from metazoan organisms fail to proliferate in the absence of appropriate growth factors. Johnston et al. have further suggested that cellular progression to a cancerous phenotype may represent a reversion of metazoan cells to a more primitive evolutionary phenotype whose “freedom to proliferate” occurs at the expense of the host's long-term survival [5]. This suggestion is supported by similarities between the behavior of cancer cells and the likely behavior of unicellular ancestors of metazoan organisms, as in both cases their growth is not subject to external regulation by other cells. Again, this phrasing also implies a freedom *not to* proliferate. However, as pointed out by Soto and Sonnenschein [4], that freedom does not seem to be enjoyed even by modern unicellular eukaryotes. If the default state of growth is truly different between unicellular and multicellular organisms, then it follows that a profound change in the default state of cells occurred during evolution of metazoan organisms and that the change is reversed in carcinogenesis. However, there may be another, simpler explanation that requires considering the growth-promoting signaling pathways from an evolutionary perspective.

The earliest cells would necessarily have been required to be independently growing entities limited externally only by the supply of resources and internally by their capacity to proliferate. As also argued by Johnston et al. [5], millennia of selective pressures in unicellular ancestors of modern eukaryotes would have favored those that were able to divide most rapidly. In that way, evolution may have led to the development of early cells as organisms designed primarily or entirely to proliferate at the maximum possible rate. During the evolution of metazoans, it seems highly unlikely that complex, ligand-dependent signaling pathways emerged de novo as a requirement for growth. Rather, the most straightforward explanation for their appearance may be that they evolved from growth-related processes that already occurred in a ligand-independent manner in their unicellular ancestors. There may be any number of ways that could have been accomplished. One such example may be presented by the membrane-associated Ras protein and its associated signaling pathways, which play a vital role in cell cycle progression of both unicellular organisms and cells of metazoans. At least in some unicellular organisms such as yeast, regulation of Ras does not involve receptors for extracellular ligands, but rather involves intrinsic signals relating to stress and metabolism [6]. However, in cells of most or all metazoan organisms, Ras acts as a downstream effector for growth factor receptor signaling [7]. Thus, in metazoan cells, growth factor signaling pathways may have arisen by co-opting processes that occurred constitutively in their unicellular ancestors. In that sense, ligand binding may not actually stimulate growth but instead permit it by completing an interrupted circuit, in the same way that turning on a light switch permits the flow of electricity to light a bulb. It is possible to envision other scenarios that might also have led to the appropriation and repurposing of intracellular processes to become part of other ligand-responsive signaling pathways in metazoans. In the assessment of any such potential scenarios, it may be particularly instructive to analyze the structure and functions of signaling pathway components identified in unicellular organisms that are closely related to metazoans [8].

If this proposal is correct, then the function of ligand-stimulated signaling pathways could be viewed not as a system wherein growth factors can be added to promote growth but rather as a system in which growth factors can be withheld to control growth. Thus, the quiescence of cultured metazoan cells in the absence of growth factors would not reflect a passive lack of growth stimulation but rather an active process of growth inhibition. Furthermore, such a model would imply that oncogenes, which are considered stimulators of growth, actually function to permit growth by overcoming limitations imposed during the transition to multicellularity.

Although this concept does not argue against an important role for genetic changes within cells during progression to cancer, as proposed in the SMT, it does support the idea of proliferation as a default state of cells, as proposed in TOFT [1, 3, 4]. It further suggests that gene products that appear to “promote” growth actually act to reveal the cell's innate tendency to grow. Thus, this proposal calls into question the gas pedal and brake analogy for cell growth. If it is valid, then perhaps a better comparison would be a soapbox racer rolling down a hillside pathway. In this analogy, the pull of gravity would represent the innate tendency to grow. Transient and reversible growth-inhibitory processes (e.g. absence of ligand) could be incorporated into this model as brakes that could be applied by the driver. Mechanisms to permanently halt growth are readily incorporated into such an analogy by invoking runaway lanes on one side of the pathway into which the racer can be steered and brought to rest off the main pathway (terminal differentiation), and by invoking a steep cliff face parallel to the pathway on the other side over which the racer could be steered (apoptosis). This analogy would also suggest that at least some limited cell cycle progression would be required for cell death, a proposal that is consistent with observations of cell-cycle progression in many apoptotic cells [9]. Although superficially this is a more complicated analogy than the gas pedal and brake, this model is still approachable and more readily accounts for the various potential fates of the cell. In addition, this concept may better account for the evolutionary emergence of growth-factor dependence in cells of metazoans.

## References

[1] Vaux DL (2011). In defense of the somatic mutation theory of cancer. BioEssays.

[2] Vogelstein B, Kinzler KW (2004). Cancer genes and the pathways they control. Nat Med.

[3] Thomas D, Moore A (2011). Counterpoints in cancer: The somatic mutation theory under attack. BioEssays.

[4] Soto AM, Sonnenschein C (2011). The tissue organization field theory of cancer: A testable replacement for the somatic mutation theory. BioEssays.

[5] Johnston RN, Pai SB, Pai RB (1992). The origin of the cancer cell: oncogeny reverses phylogeny. Biochem Cell Biol.

[6] Tamanoi F (2011). Ras signaling in yeast. Genes Cancer.

[7] Margolis B, Skolnik EY (1994). Activation of Ras by receptor tyrosine kinases. J Am Soc Nephrol.

[8] King N, Westbrook MJ, Young SL, Kuo A (2008). The genome of the choanoflagellate *Monosiga brevicollis* and the origin of metazoans. Nature.

[9] Fotedar R, Diederich L, Fotedar A (1996). Apoptosis and the cell cycle. Prog Cell Cycle Res.

